# DanMAX – a beamline for materials science using powder X-ray diffraction, total scattering, and full-field tomographic imaging

**DOI:** 10.1107/S1600577526009471

**Published:** 2026-10-08

**Authors:** Mads Ry Vogel Jørgensen, Innokenty Kantor, Frederik Holm Gjørup, Daniel Riskov Sørensen, Thorbjørn Erik Køppen Christensen, Hari Narayanan Vasavan, Arash Panahifar, Silvia Forcat, Erik Bergbäck Knudsen, Marcelo J. P. Alcocer, Felix Engelmann, Henrik Enquist, Bo Brummerstedt Iversen, Yimeng Li, Henning Friis Poulsen, Peter Sondhauss, Hammed Tarawneh, Clemens Weninger

**Affiliations:** a Department of Chemistry Aarhus University Langelandsgade 140 8000Aarhus C Denmark; b MAX IV Laboratory Lund University Fotongatan 2 Lund224 84 Sweden; c Department of Physics Technical University of Denmark Fysikvej 310 2800Lyngby Denmark; d Department of Applied Mathematics and Computer Science Technical University of Denmark Bygning 324 2800Lyngby Denmark; Brazilian Synchrotron Light Laboratory, Brazil

**Keywords:** materials science, *in situ*diffraction, total scattering, µXRD mapping

## Abstract

DanMAX is a hard X-ray materials science beamline at the ultra-low emittance 3 GeV storage ring at the MAX IV laboratory. It is designed for performing *in situ* and *in operando* studies using powder X-ray diffraction and full-field imaging. The beamline is equipped with an in-vacuum undulator source providing photons in the energy range 15–35 keV. The beam can be focused using compound refractive lenses, thus providing a variable beam size range from approximately 15 µm to 1.2 mm (FWHM) that can be changed in seconds. This paper presents the X-ray optics, discusses its performance, and shows the first endstation instrument for powder X-ray diffraction, available sample environments, and the surrounding infrastructure. The powder diffraction instrument is equipped with a CdTe hybrid pixel detector for powder X-ray diffraction and total scattering, as well as an energy-resolved silicon drift detector for X-ray fluorescence experiments. The instrument and infrastructure are designed to be highly flexible to accommodate various sample environments for *in situ* and *in operando* experiments.

## Abstract 2

The DanMAX beamline at the MAX IV laboratory is designed for *in situ* and *in operando* studies using powder X-ray diffraction, total scattering, and full-field imaging in the 15–35 keV energy range.

## 1. Scope of the DanMAX beamline

Material properties are intrinsically linked to their structures at various scales, from the atomic to the microstructural level. Understanding these relationships is fundamental to material science, as it enables designing and optimizing materials with desired properties for specific applications. To fully understand the material properties and performance, it is pivotal to understand the structural mechanisms governing the properties during operation. This entails probing the structure under conditions as close to the processing or operational conditions as possible.

The DanMAX beamline started user operation in late 2021 and is located at achromat 4 of the 3 GeV storage ring at the MAX IV laboratory. The storage ring is the first realization of a fourth-generation synchrotron light source offering unparalleled brightness and tunability for probing materials at atomic and molecular levels (Robert *et al.*, 2023 ▸; Tavares *et al.*, 2018 ▸).

This paper describes the beamline, including the X-ray optics, and its performance. We also present the first of three planned instruments. This first instrument is designed for powder X-ray diffraction (PXRD) and total scattering (TS), and is presented along with available sample environments and infrastructure. The full-field microtomography instrument has been installed at the beamline and entered the user program in late 2024. This instrument and a high angular resolution powder diffractometer with a Si-based 1D position-sensitive detector, currently under construction, will be described in future publications.

## 2. Technical design

**Figure 1:**
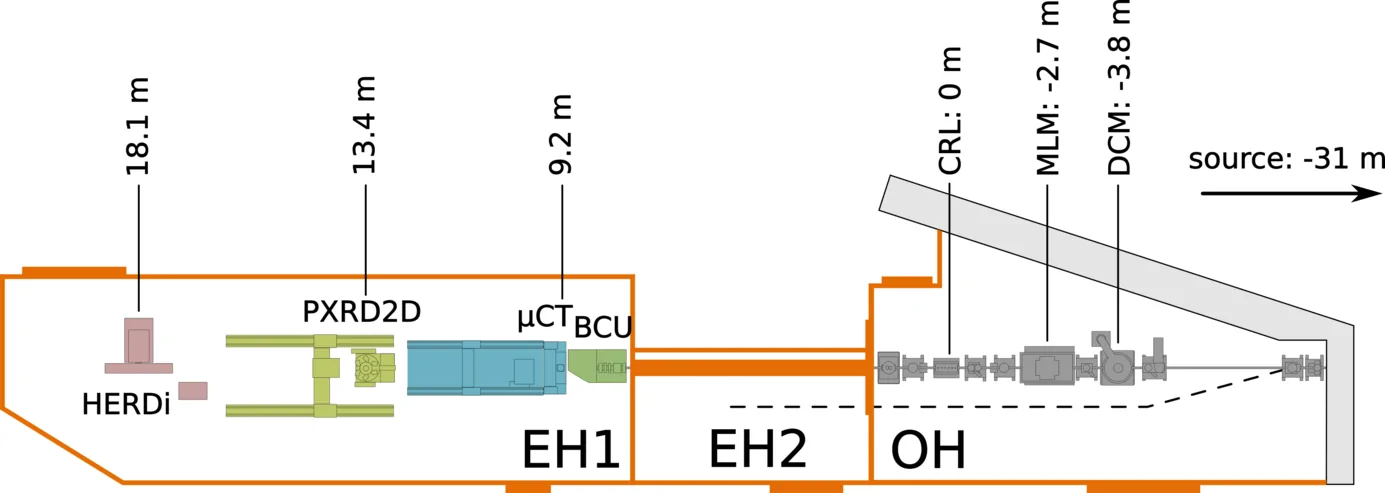
Beamline layout. DCM is a double crystal Si(111) monochromator, and MLM is the multilayer monochromator. OH is the optical hutch, EH1 is the main DanMAX experimental hutch. EH2 is a SINCRYS side-branch beamline (dashed line, under construction). Distances from the CRL transfocator (center of the undulator) are shown for straightforward calculation of demagnification at the three instruments. BCU – beam conditioning unit; µCT – micro-CT imaging instrument; PXRD2D – an area detector powder X-ray diffraction instrument; HERDi – a high resolution PXRD instrument (under construction).

The beamline consists of an optical hutch and a single large experimental hutch where all three instruments are placed in series, see Fig. 1 ▸. The temperature in the optics and experimental hutches is controlled to 0.2 K (peak to peak) over a 24 h period. Point extraction process ventilation is available for all instruments in cases where fumes may develop or be released during the experiment. A second experimental hutch, EH2, is placed between the optics hutch and the large experimental hutch. This experimental hutch will be used for a side-branch beamline, SINCRYS, for single-crystal X-ray diffraction experiments on microcrystals. The side station will only be able to accept a narrow range of energies, ∼20–23 keV, from the 7th to 9th harmonic. The beam for the side branch is extracted using the 111 reflection from a 100 µm-thick diamond crystal in horizontal Bragg geometry. This solution allows both EH1 and EH2 to operate in parallel. The energy and energy range are limited mainly by available space in the optics hutch. The side-branched beamline is currently under construction and will be described in a future publication. The science case for the instruments in EH1 requires the full energy range (15–35 keV) of the beamline, and thus it was decided to build all instruments in series on the main branch.

### 2.1. X-ray source

The X-ray source is a 3 m in-vacuum hybrid undulator with a 16 mm period length (IVU16, Hitachi Metals Ltd, Japan). The IVU16 is designed to operate with a minimum gap of 4.0 mm, corresponding to a *K*_max_ of 1.66, providing an X-ray energy range of 12–35 keV using the 5th to 13th harmonics (Huang *et al.*, 2021 ▸). The as-built IVU reaches a *K* value of 1.67 at a gap of 4.4 mm, and, thus, the minimum gap is restricted to 4.4 mm. The source characteristics of the MAX IV storage ring, see Table 1 ▸, and the long, 187 period, IVU16, allow minimization of the heat load on the primary optics without sacrificing the usable monochromatic photon flux by limiting the allowed divergence in the front-end (FE; Toyama Co. Ltd, Japan). A movable aperture in the FE defines an acceptance cone of 35 µrad × 35 µrad (700 µm × 700 µm at approximately 21 m from the source), and reduces the maximum power transmitted through the FE from 10.4 kW to approximately 130 W (at 500 mA storage ring current). The heat load is further reduced to approximately 65 W using two single-crystal diamond (CVD) high-pass filters with a combined thickness of 1.0 mm in the beam path.

### 2.2. Primary optics

**Figure 2:**
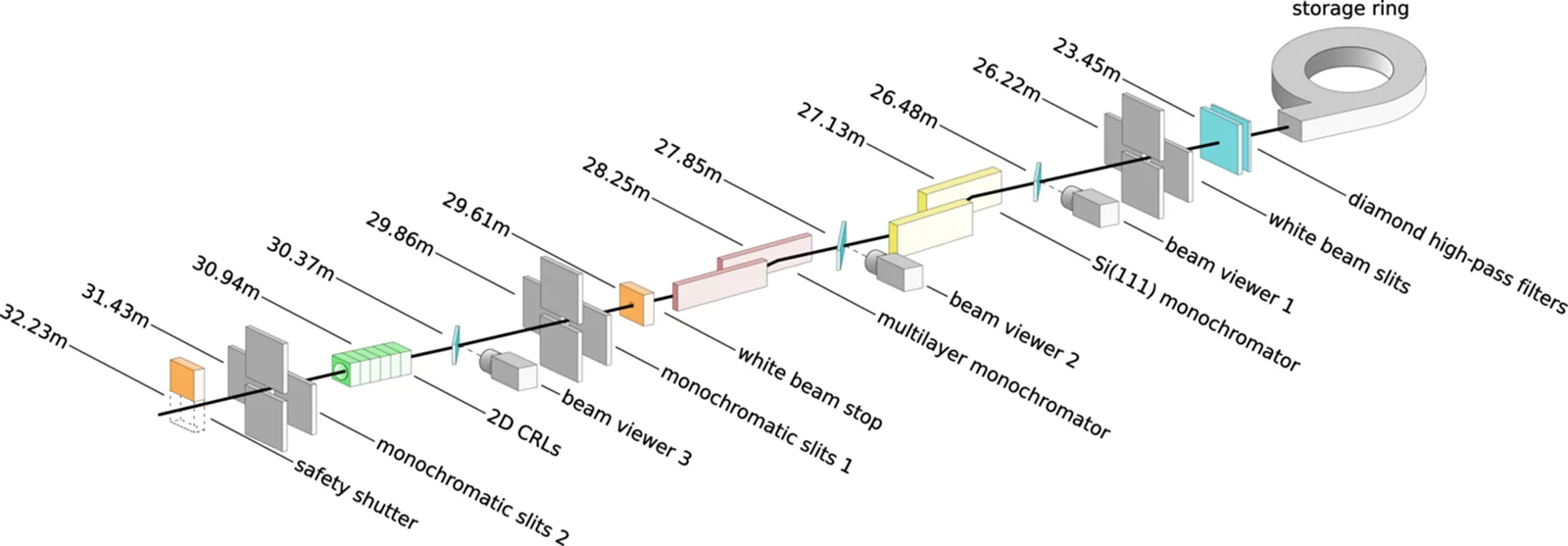
Overview of X-ray optics of DanMAX. Distances shown are from the center of the undulator.

The primary optics are all located in the optics hutch (OH in Fig. 1 ▸) and are designed to condition the beam energy, band-pass, divergence, and beam size suitable for each different experiment. A schematic layout of the optical components is shown in Fig. 2 ▸. The beamline is optimized for the energy range 15–35 keV, but all components can work down to 12 keV.

#### 2.2.1. Double crystal monochromator

The first optical element is a cryocooled Si(111) horizontally deflecting double crystal monochromator (DCM; FMB Oxford Ltd, UK). The horizontal geometry increases the stability at the expense of a small intensity reduction, up to ∼13% at 15 keV, due to p-polarization of the beam (Kristiansen *et al.*, 2016 ▸). The monochromator is designed to operate at a fixed 4 mm or 10 mm offset, depending on operation mode (see infra). Both crystals are side-cooled via copper blocks. Pressurized liquid nitro­gen is circulated directly through the first crystal blocks, whereas the second crystal blocks are cooled via copper braids connected to the first crystal blocks. The weaker coupling to the second crystal provides sufficient cooling while minimizing the vibration of the second crystal.

#### 2.2.2. Double multilayer monochromator

For even higher flux, it is possible to omit the DCM and instead use a wide bandpass monochromator consisting of two multilayer mirrors in a horizontally deflecting fixed exit configuration (MLM; FMB Oxford Ltd, UK). The two flat silicon (100) substrates (Coastline Optics, USA) each have two multilayer stripes (coated by Rigaku, Japan) with different bandpasses, see Table 1 ▸. Both stripes cover the full energy range from 12 to 35 keV. The mirrors are side-cooled through copper blocks. The first mirror is water-cooled directly through the block, whereas the second is cooled through an extension of the copper blocks protruding into a water-cooled trough with a eutectic melt of gallium, indium, and tin.

The MLM can be operated independently with a 10 mm offset as described above or be used with a 6 mm offset in combination with the DCM in 4 mm offset mode. Using both monochromators in series leads to highly effective higher harmonic rejection as the refractive deflection in the MLM does not transmit the third harmonic from the DCM, leading to remarkably high attenuation of the higher harmonics. Higher harmonic contamination is normally not a problem and is barely detectable in the PXRD data at 15 keV for highly crystalline powders. The experimentally measured ratio of the third to first harmonics ratio at 15 keV is of the order of 10^−5^, closely matching ray-tracing simulations. However, higher harmonic contamination can become an issue if the beam is heavily attenuated, as the fundamental energy is attenuated while the higher harmonics are much less affected, thereby increasing the higher harmonic ratio. In these cases, it is possible to use both the DCM and MLM in combination or alternatively open the IVU to use a higher undulator harmonic and thus lower the total flux to avoid the need for attenuation.

#### 2.2.3. Transfocator

The beam can be focused using a transfocator (FMB Oxford Ltd, UK) with up to 50 2D parabolic beryllium compound refractive lenses (CRLs; RXOPTICS GmbH & Co. KG, Germany). The curvature radius of the lenses is 200 µm with a geometric aperture of 880 µm. The CRLs are placed in six cassettes, allowing insertions of any number of lenses from 0 to 50, Table 1 ▸. The cassettes are inserted into the beam using pneumatic actuators and aligned using a precision-machined V-grove at a fixed position along the beam. The lens cassettes are connected to the pneumatic actuator via copper braids to provide thermal contact for cooling the lenses. The insertion and extraction of the lens cassettes is fast, ∼1 s, and no alignment is needed after changing the number of lenses, thus providing a very flexible solution for changing the beam size. The size of the focal point can be adjusted by changing photon energy. This can be particularly useful for the lower photon energies (15–25 keV) if the exact photon energy is not critical for the experiment.

#### 2.2.4. Slits, attenuators, and beam diagnostics

The beamline is equipped with three sets of slits in the optics hutch and one for each instrument in the experimental hutch. The first (white beam) slits, positioned upstream of the DCM, limit the acceptance of the monochromators and are designed to handle the full pink beam intensity. Two monochromatic slits are placed downstream of the MLM and the transfocator, respectively. These slits are designed to handle the full intensity of the MLM beam. In normal operation, all three slits are left wide open as the FE defines an acceptable beam for all optical components. Unless the beam is focused using the CRLs, the beam size will normally be determined by an in-air slit (AT-F7-AIR, JJ X-ray, Denmark) on the beam conditioning unit (BCU) at the upstream end of the experimental hutch.

Three retractable beam viewers (BVs; FMB Oxford, UK) are positioned in the beam path in the optics hutch. The first BV is placed immediately downstream of the white beam slits and can thus be used to verify the alignment of both FE apertures and the white beam slit. The second BV is placed between the DCM and the MLM to verify both the location of the pink beam or the monochromatic beam from the DCM. The third BV is located just upstream of the transfocator and can image the monochromatic beam from either the DCM or the MLM. All BVs image the beam using fluorescence from a diamond screen detected by CMOS cameras. The first and third BVs use 200 µm-thick polished single-crystal CVD diamond screens to minimize perturbation of the wavefront of the beam. The second BV uses a polycrystalline CVD diamond due to its much larger size of 33 mm in the horizontal direction. All BVs are also equipped with a diode positioned at a 90° scattering angle in the horizontal plane, which can be used as intensity monitors. The two upstream BVs are usually not inserted during experiments but are mainly used for alignment or troubleshooting. The third BV, imaging the monochromatic beam, is usually inserted during PXRD and scattering experiments. For imaging experiments, all BVs are usually extracted to preserve the wavefront. An additional compact and portable beam viewer, based on imaging fluorescence from a Ce:YAG screen, is available in the experimental hutch, *e.g.* for beam alignment and focusing.

A white beam stop prevents the direct white beam from entering the experimental hutch. Thus, only a monochromatic beam (narrow or wide bandpass) can be used in experiments.

A set of attenuating Ni and W foils of different thicknesses is located on the BCU. Using different metals and foil thicknesses, variable attenuation is possible across the full energy range. A set of reference foils (Zr, Ag, Te) are also available for monochromator energy calibration verification via absorption edge scans. A fast experimental shutter, ∼10 ms opening time, is also located on the BCU, along with the retractable alignment laser. The BCU also allows mounting of additional collimating and secondary focusing optics, significantly expanding the flexibility, particularly for the imaging instrument. These capabilities will be elaborated in a future paper on the full-field imaging instrument.

## 3. *In situ* powder X-ray diffraction endstation

**Figure 3:**
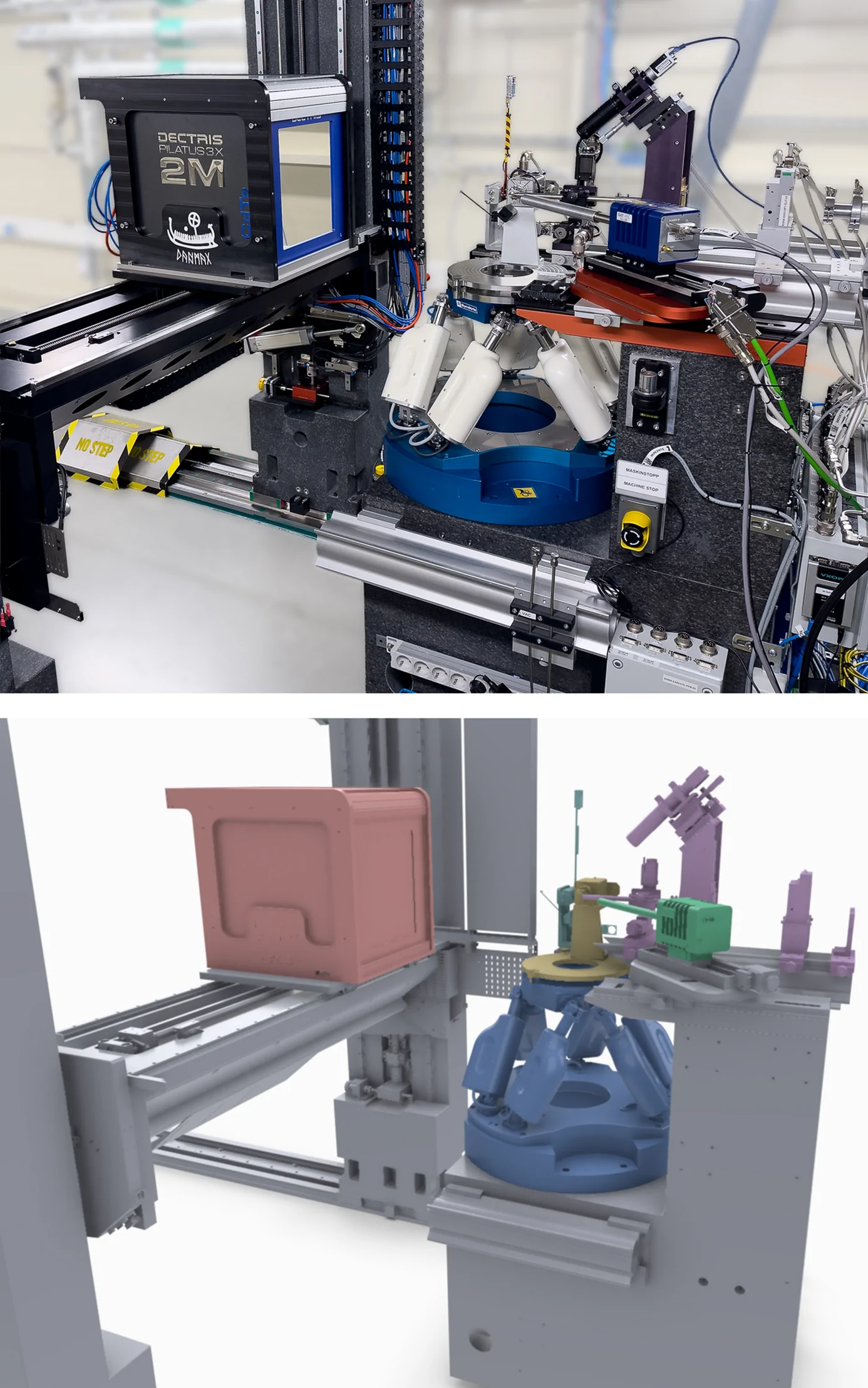
The PXRD2D instrument at the DanMAX beamline. A photograph of the instrument is shown at the top, and a rendering with color-coded components is shown at the bottom for clarity. The beam enters from the right in the picture and propagates to the left. Red: area detector; blue: hexapod; green: XRF detector; dark green: beam stop and in-beam diode; purple: slits, pinhole, and microscope; yellow: sample environment, in this case a capillary spinner. The support table and detector gantry in granite are shown in gray.

The first of the three endstation instruments to be commissioned at DanMAX is the powder X-ray diffraction instrument equipped with an area detector (PXRD2D). The instrument, see Fig. 3 ▸, is designed to be flexible and accommodate a wide range of sample environments. The instrument is placed on a stable granite block with two separate levels. The main sample stage is a heavy-duty hexapod with stepper motors and absolute encoders (Breva ST, Symétrie, France) placed on the lower level. It provides six degrees of freedom, capable of carrying a load of up to 200 kg with sub-µm repeatability. Hexapods provide a compact and very stiff platform compared with stacked stages with the same degrees of freedom and load capacity. The hexapod also has a Ø120 mm through-hole in the mobile (upper) platform, which is often used for routing cables from the sample environments or for, for example, a heat gun mounted on the granite below. The nominal space between the beam and the upper platform is approximately 200 ± 50 mm. A 100 mm thick removable spacer is positioned below the hexapod, increasing the available height between the beam and the upper platform up to approximately 350 mm.

To enable fast sample environment changes, a kinematic mounting system was developed and mounted on the movable platform of the hexapod. Each sample environment is built on a matching interface plate secured using thumbscrews. This system ensures that sample environments can be exchanged rapidly and reproducibly. A 3D model of the required baseplate is available via the beamline website for users interested in developing compatible sample environments. A 25 mm pitch M6 breadboard is also available for mounting of user equipment.

All other equipment on the instrument is mounted on X95 aluminium profiles (Newport Corporation, USA) for added flexibility. This includes guard slits, a motorized pinhole, a motorized beam stop, a microscope, and cameras, an I_0_ diode, and a silicon drift detector for X-ray fluorescence (see below).

### 3.1. X-ray detectors

The main detector on the instrument is a PILATUS3 X CdTe (DECTRIS AG, Switzerland) hybrid pixel area detector with a pixel size of 172 µm (Donath *et al.*, 2013 ▸). The detector has a large active area and is photon-counting, providing virtually noise-free detection. It can operate continuously at 250 Hz with full area readout and 20-bit dynamic range. The detection layer is made from CdTe, which was originally intended for high-energy applications. The benefit of using this detection layer over, for example, Si in the hard X-ray range available at DanMAX, 15–35 keV, is its near 100% quantum efficiency and that the X-rays are detected near the surface of the CdTe layer. This nearly eliminates both oblique incidence angle and parallax effects, thus improving the angular resolution (Marlton *et al.*, 2019 ▸; Chernyshov *et al.*, 2021 ▸).

The detector is placed on a large gantry, providing four degrees of freedom. It is possible to place the beam center either in the center of the detector or anywhere on the lower left quadrant (as seen from the beam), or even put the beam center outside of the detector. The sample-to-detector distance (SDD) can be as low as ∼100 mm and up to approximately 3000 mm. Placing the beam near the lower edge of the detector at 170 mm SDD leads to a *Q*_max_ of approximately 19 Å^−1^, suitable for *in situ* (and/or in solution) pair distribution function (PDF) analysis. It is possible to tilt the detector towards the sample by up to 45° to increase the *Q* range further. To guide users in finding the best detector geometry, we have developed the application *xrdPlanner* (Krause *et al.*, 2024 ▸), which makes it possible to visualize how a known sample (via a CIF file) will diffract on the detector while varying the geometry and photon energy.

**Figure 4:**
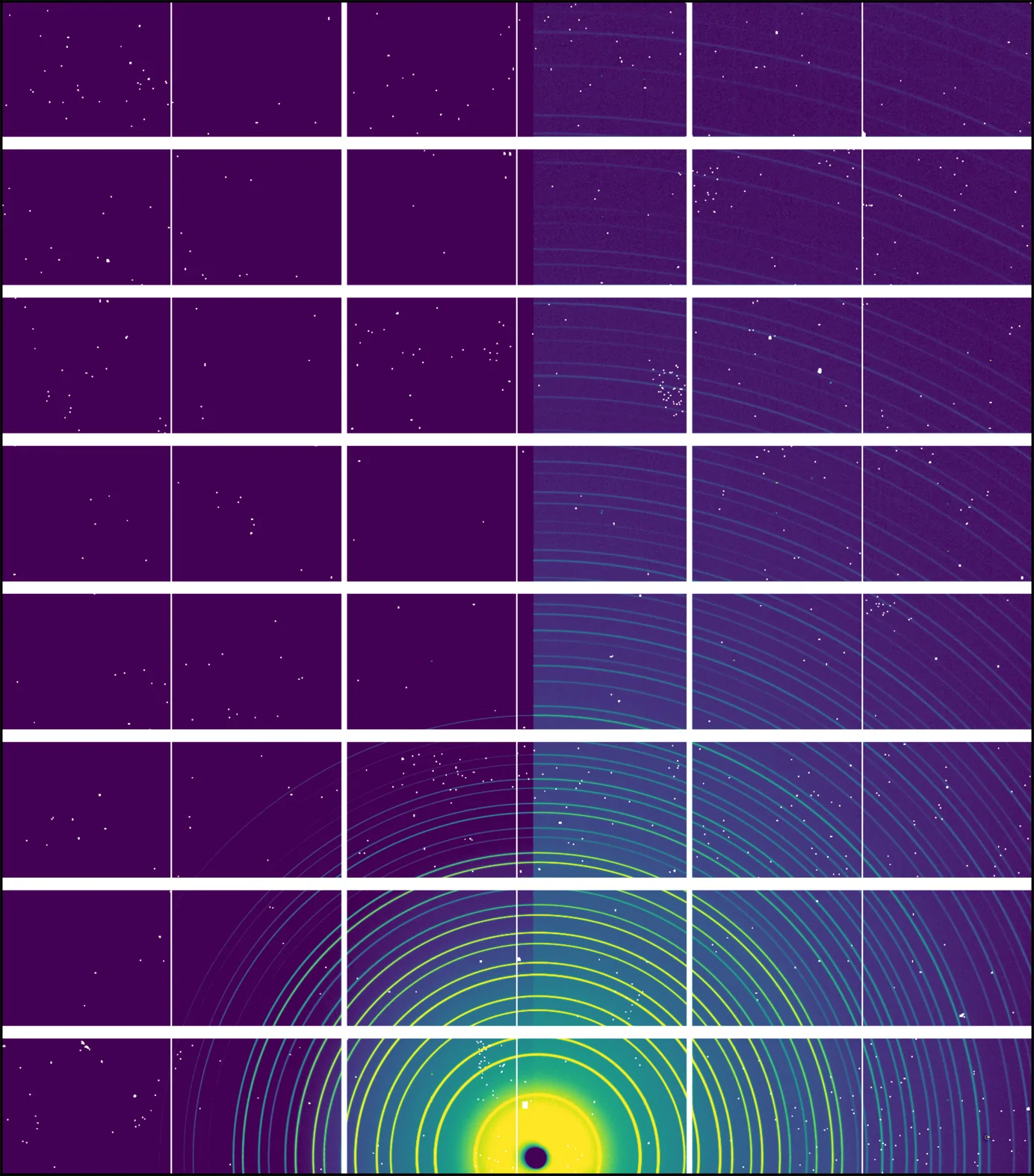
Detector image from a Si standard in a 0.2 mm capillary, comparing the raw (left) with the solid-angle and polarization corrected image (right). SSD: 135 mm; X-ray energy: 35 keV. Shown with a log scale color map.

A challenge of using a large area detector at a small SDD is the large effects of solid angle, and to a certain extent the polarization, making rings at higher scattering angles harder to identify during detector geometry calibration. Fig. 4 ▸ shows the difference in applying the solid angle and polarization corrections on the 2D diffraction image of a Si sample in the PDF geometry. To help our users, we have developed a Python wrapper around the *pyFAI-calib2* application (Kieffer *et al.*, 2020 ▸). The wrapper will use the detector gantry position to calculate the approximate solid angle and polarization corrections and apply these to the image. It will further run peak finding and geometry refinement using the command line interface of *pyFAI* (Kieffer *et al.*, 2025 ▸) before opening the corrected image in *pyFAI-calib2*. This will in many cases produce a good calibration while giving the user the option to tweak the peak finding and geometry refinement further.

To integrate the detector into the data acquisition system at MAX IV, software was developed that compresses the raw data using LZ4 bit shuffle compression (Masui *et al.*, 2015 ▸) and streams the compressed data to the centralized data storage using the ZeroMQ protocol. This software runs directly on the detector control unit (DCU) connected to the PILATUS detector. The data stream allows for live visualization of the raw detector frames and live processing of the frames in parallel with storing them in the central data storage facility at MAX IV (Bell *et al.*, 2026 ▸).

A single-element silicon drift detector (Sirius-SD, RaySpec, UK) is available for X-ray fluorescence (XRF) experiments. The raw signal is processed into an energy-resolved histogram using a pulse processor (Xspress3, Quantum Detectors, UK). The XRF detector is normally used in a backscattering geometry in the horizontal plane, at a 20–50° angle to the incident X-ray beam. The detector is placed in air but has routinely been used to detect elements as light as potassium. While the detector is normally used to collect data from the sample, it can also be used as a transmission detector by measuring the fluorescence signal from the beam stop (Birkedal *et al.*, 2024 ▸). To ensure the beamstop signal does not interfere with the sample signal, the beamstop can be filled with different metals.

One of the aims for the beamline is to provide relevant live feedback to the user, ideally leading to more successful experiments. Live viewers visualizing the data streams are implemented for both the area detector and the XRF detector. An additional aim is that users should leave the facility with azimuthally integrated data (or reconstructed data for tomographic experiments) to lower the barrier of starting the analysis, which should ideally lead to faster publication of the results. The raw detector frames are azimuthally integrated using a Python and C++ implementation of the MATFRAIA algorithm (Jensen *et al.*, 2022 ▸). The azimuthal integration is performed live on the data stream and has been used at detector speeds up to several kHz (Donath *et al.*, 2025 ▸). The integration is always performed over the whole (masked) detector area and optionally also in user-specified azimuthal bins for, for example, analysis of crystallographic texture. The integrated data are available for visualization during the experiment to provide feedback to the operators. The azimuthally integrated data are written in the NXAzint format containing all relevant processing metadata (Jørgensen *et al.*, 2026 ▸).

The raw and integrated data are written to HDF5 data files, which are stored on a MAX IV centralized GPFS file system using solid-state drives, ensuring consistent I/O speeds higher than the maximum data rate of the detectors, allowing continuous operation of the detectors at full frame rates (Bell *et al.*, 2026 ▸). As soon as a scan finishes, the data are available for analysis both on-site and for remote users.

To aid users in evaluating the data, we have developed a library of Python-based *Jupyter* notebooks (Kluyver *et al.*, 2016 ▸) that are copied to the user’s directory when an experiment is started. The user can run these through MAX IV’s Jupyterhub, which is accessed via a web browser. By using MAX IV computing hardware, no software installation is needed, and users benefit from a fast connection to the storage system. It further allows off-site participants to aid in evaluating and analyzing the data. Examples of the functionality available in the notebooks are simple visualization, *i.e.* preparing waterfall plots as a function of time or metadata channels; *e.g.* temperature, plotting Pearson autocorrelation maps to locate phase transitions, and non-negative matrix factorization for multicomponent systems. More advanced features include peak and XRF spectrum fitting as a function of time or metadata channels, and preparing 2D maps based on either µXRD or µXRF data, *etc*. Visualization of the PXRD data, in the NXazint format, is also possible in the program *plaid* developed at the beamline (Gjørup, 2026 ▸).

### 3.2. Control system

MAX IV uses *Tango* (Chaize *et al.*, 1999 ▸; Juerges *et al.*, 2023 ▸) as the general control system, and most equipment at DanMAX is controlled via *Tango* device servers. All stepper motors are controlled using IcePAP modules (Janvier *et al.*, 2013 ▸). The experimental orchestration is performed using *Sardana* (Coutinho *et al.*, 2011 ▸), which handles the arming of detectors and file writing of the low data-rate channels, *e.g.* counters and temperatures, and extended metadata. As described above, high-data-rate detector data are streamed directly to receiving software for analysis and storage.

Central to the experimental orchestration at DanMAX is a PandABox (Zhang *et al.*, 2017 ▸). This device is built on a highly configurable FPGA processing unit that can be configured using a web interface. Here, it sends TTL trigger/gate signals to all detectors at the beamline and controls the fast experimental shutter. The PandABox can also record encoder signals, making it ideal for continuous scans for techniques such as µXRD mapping and XRD-CT, where traditional step scanning is prohibitively slow. The PandABox is also used to record TTL signals that can be used to accurately align data sets from user-provided sample environments during asynchronous scans.

Photoinduced currents on Si diodes for beam intensity monitoring, flux measurements, and data normalization are recorded using electrometers developed in collaboration between ALBA-CELLS Synchrotron and MAX IV (Avila-Abellan *et al.*, 2017 ▸).

## 4. Experimental infrastructure

### 4.1. Sample environments

Various devices are available to control the sample position and environment during experiments. All the devices listed in Table 2 ▸, described below, are currently available in the general user program.

To facilitate easy installation of users’ own equipment, several patch panels with RJ45, BNC, and SUB-D connectors connect the experimental station with the control room. There are also patch panels available for stepper motors (including encoders) so users can connect and control their own equipment from the beamline control systems. Finally, two user-operated chicanes are installed in the experimental hutch, making it fast and convenient to pull other cables and tubes if needed.

Several mechanical stages are available for sample positioning and manipulation. A commonly used stage is a sample spinner for capillary samples driven by a stepper motor with a rotation speed of up to 5 Hz. The spinner can be configured for either a horizontal or vertical sample axis and uses a manual goniometer head (1005, Huber Diffraktionstechnik GmbH, Germany) to center the capillary. The spinner itself is aligned to the X-ray beam using the hexapod described above.

Linear stages with encoders are available in *XY* configurations to facilitate continuous Cartesian scans (a.k.a. fly scanning). Two configurations are used: high speed and accuracy (LIMES 84N-IMS, OWIS GmbH, Germany) for µXRD+XRF mapping and higher load capacity (8MT50, STANDA, LT) for heavier samples, up to approximately 5 kg, and XRD-CT (Stock *et al.*, 2008 ▸). The high speed and accuracy stages have a repeatability of less than 0.5 µm and a maximum velocity of 20 mm s^−1^. The high load stages have a repeatability of 2.5 µm and a maximum velocity of 10 mm s^−1^. For XRD-CT experiments, the stages are combined with a rotation table (408, Huber Diffraktionstechnik GmbH, Germany), or an air-bearing rotation stage (RT150ST, LAB Motion Systems, Belgium). The stages are all equipped with a kinematic base (M-BKL-4, Newport Corporation, USA) to facilitate easy and reproducible sample changes.

#### 4.1.1. Temperature control

Sample temperature is one of the external parameters often examined using PXRD and TS. For low-temperature applications, a Cryostream 800 Plus (Oxford Cryptosystems, UK) covers a temperature interval from 80 to 500 K with a 0.1 K temperature stability. The maximum temperature ramping rate is 300 K h^−1^ (5 K min^−1^). The high-temperature range, room temperature to 1000 K, is covered by a hot air blower (HiHeat type A 500 W, HyBec, Japan) controlled by a Lake Shore 336 temperature controller (Lake Shore Cryotronics, Inc., USA) and powered by a separate power supply (PSI 5080, EA Elektro-Automatik GmbH & Co. KG, Germany). The temperature stability is approximately 5 K, and the temperature can be ramped at up to 100 K min^−1^ (Roelsgaard *et al.*, 2023 ▸). Faster heating rates are possible without a controlled ramp. Both devices are integrated into the control system, and temperature data are recorded in the data files. High photon flux allows for continuous data collection during temperature ramping, making variable temperature not only faster than collecting X-ray data at predetermined temperature points but also capable of detecting exact transition temperatures. In these asynchronous scans, temperature data are continuously recorded at the same rate as the X-ray data.

For higher temperatures and especially higher heating rates, the bespoke ARΩS furnace can be used. The furnace uses ohmic heating of a carbon fiber felt surrounding the sample and is designed to obtain texture information from sample pellets during sintering (Shyam *et al.*, 2023 ▸; Laursen *et al.*, 2024 ▸). It operates in a vacuum or inert gas environment and can heat up to 1500 K at controlled heating rates up to more than 150 K s^−1^.

#### 4.1.2. Batteries

The chemistry of batteries is inherently out of equilibrium, and thus must be studied *in operando* to understand the structural changes as they occur. To facilitate *in operando* studies, the beamline is equipped with a 14-channel potentiostat (VMP3, Bio-Logic SAS, France) that can simultaneously and independently charge and discharge up to 14 batteries in parallel while recording electrochemical data. Twelve standard channels provide voltages up to ±10 V (adjustable between −20 to +20 V), and current between 400 mA and 10 µA with a resolution of 0.760 nA. Two low current channels provide currents down to 1 nA with a resolution of 76 fA. The potentiostat is controlled via the proprietary *EC-Lab* software (Bio-Logic SAS, France), but absolute time stamps in both the X-ray and electrochemistry data files make it simple to combine the data during analysis.

The most commonly used battery cell at the beamline is the AMPIX cell (Borkiewicz *et al.*, 2012 ▸), where 12 cells are available at the beamline. An 8 AMPIX cell holder compatible with the high-load *XY* stages is available, enabling parallel X-ray diffraction experiments on up to eight cells. X-ray data are measured sequentially on the cells while the electrochemistry is controlled in parallel. In this way, it is possible to get X-ray diffraction data on each cell approximately every 30–60 s. Similar holders are also available for up to six pouch cells (Gustafsson *et al.*, 2021 ▸) and twelve CR2032 cells with holes through the steel casings.

#### 4.1.3. Solvothermal reactor

A capillary reactor for solvothermal experiments is available for studying, for example, crystallization of nanoparticles using TS and PDF analysis or PXRD. The reactor is based on a modular system where the fused silica capillary is held in a standardized sample holder. Ten holders are available at the beamline, allowing off-line preparation of precursors in advance, enabling effective use of the beam time. The capillary is pressurized using an HPLC pump and heated using the hot air gun described above. A polycarbonate splash box protects the surrounding equipment from potential ruptures of the capillaries. A Kapton-covered cutout towards the detector ensures that the diffracted X-ray reaches the detector with minimum attenuation. A full description of the reactor design and performance has been published recently (Roelsgaard *et al.*, 2023 ▸).

#### 4.1.4. High-pressure research infrastructure

The high photon flux and high energy of the focused beam (using CRLs) allows for high-pressure materials research using diamond anvil cells (DACs). The energy used for these experiments is often in the range 33–35 keV. The minimum focused beam size at the PXRD2D instrument is approximately 15 µm × 60 µm. This beam size necessitates using a relatively large cullet and gasket aperture in the DAC, to reduce scattering from the gasket material. The larger cullet size limits the maximum pressure that can be reached. To further reduce the size of the beam, a slit can be mounted onto the final pinhole. This reduces the beam size to approximately 10 µm × 10 µm at the expense of flux loss. The slit does introduce parasitic scattering; however, the DAC effectively blocks this signal. In this configuration, pressures up to approximately 50–70 GPa are routinely attainable.

A dedicated mechanical stage has been designed to facilitate rapid alignment of the DAC sample chamber in the focused beam. The setup is placed on top of the hexapod and consists of a rotation stage (with a vertical rotation axis, omega) and translations perpendicular to the omega axis (*XZ*). The *X*-axis is configured for continuous scanning, drastically reducing the time needed for alignment. A bespoke graphical user interface supports the alignment, where experimental X-ray transmission data are fitted, and alignment corrections are automatically applied. The cells are attached using kinematic bases to facilitate reproducible positioning on the stage.

The pressure of the DACs can either be controlled manually by adjusting the screws providing the compression force or by a gas membrane compressing the two diamond anvils. The pressure in the gas membranes is controlled using a PACE 5000 controller (Druck, USA) capable of ramping the pressure up to 200 bar for dynamic high-pressure experiments. During dynamic ramping experiments, the pressure is monitored using an internal pressure calibrant, while an off-line optical micro-spectrometer setup and ruby spheres are used for manual calibration of the pressure. A spark erosion machine is available at the beamline for gasket preparation.

#### 4.1.5. Gases and humidity

A gas panel capable of mixing two gases at pressures up to 200 bar is installed in the experimental hutch. The valves on the panel are remote-controlled and support both static experiments and experiments under a gas flow. A vacuum pump is connected to the panel and, as such, both the sample and the panel can be purged. The system is approved for non-toxic and non-flammable gases only. A low concentration of H_2_ in an inert gas, *e.g.* 2% H_2_ in Ar, can be used as a reducing atmosphere. The system has also been approved for using CO_2_. Toxic and flammable gases are handled using a mobile gas cabinet that can be integrated with the beamline ventilation system.

Controlling the humidity of a sample is important in several fields, *e.g.* pharmaceutical and food science. A humidifier (P-2, Cellkraft, Sweden) can provide a flow rate from 0 to 2 L min^−1^ with 0–100% relative humidity at ambient output pressure from room temperature (RT) to 125°C. The humidifier is usually operated using nitro­gen gas.

### 4.2. Laboratory equipment

Simple sample preparation, *e.g.* filling capillaries with non-toxic samples, is possible at the beamline. Here, a stereo microscope with a digital camera and encoded zoom (SZX16, Olympus Corporation, Japan) is available. Additional equipment, *e.g.* (vacuum) ovens, scales, and a 10 ton press, are also available.

A chemistry laboratory, shared with other MAX IV beamlines, is located adjacent to the DanMAX. Fume hoods can be used for the preparation of precursors and samples. Within the lab, an argon-filled glove box is also available to users. The glove box is equipped with an activated carbon filter to facilitate work with solvents, for example battery cell preparation. Other chemistry laboratories at the facility provide other capabilities, *e.g.* grinding/polishing of samples.

## 5. Beamline performance

The beamline optics were designed to optimize ease of operation, *i.e.* to allow easy adjustment of energy, bandpass, and focus size. This means that the horizontal offset of the monochromatic beam is always 10 mm, regardless of the monochromator configuration. Focusing is performed on-axis using CRLs, thus avoiding mirrors that take more effort to align. When no focusing is needed, avoiding mirrors altogether also helps preserve the wavefront, yielding a smooth, flat field for phase contrast imaging.

The Bragg angle of the monochromator (DCM) is monitored using an accurate encoder and is routinely calibrated using *K*-edges of a range of reference metals to an accuracy of 2–5 eV. Energy changes are performed across the full energy range in a matter of minutes. This is routinely done by users during some experiments and does not require realignment.

The thermal stability of the DCM is easily monitored using the control software. No active control is performed, and the beam stability relies on the passive stability of the monochromator. After thermal equilibrium is achieved, beam intensity fluctuations are primarily due to top-up in the storage ring. Top-up injections occur every 10 min and are about 1% of the total current. The machine and/or beamline introduce an additional fluctuation of about 0.2% intensity measured at the sample position. While complete thermal equilibrium between the two crystals is reached only after a couple of hours, experiments can be conducted within a few minutes of changing the photon energy. Rocking curve (second crystal pitch) scans, fitting, and pitch correction are fully automated and can be performed by the user with a single click. This procedure is only needed before thermal equilibrium in the DCM has been reached. Once thermally equilibrated, the pitch remains stable for days.

**Figure 5:**
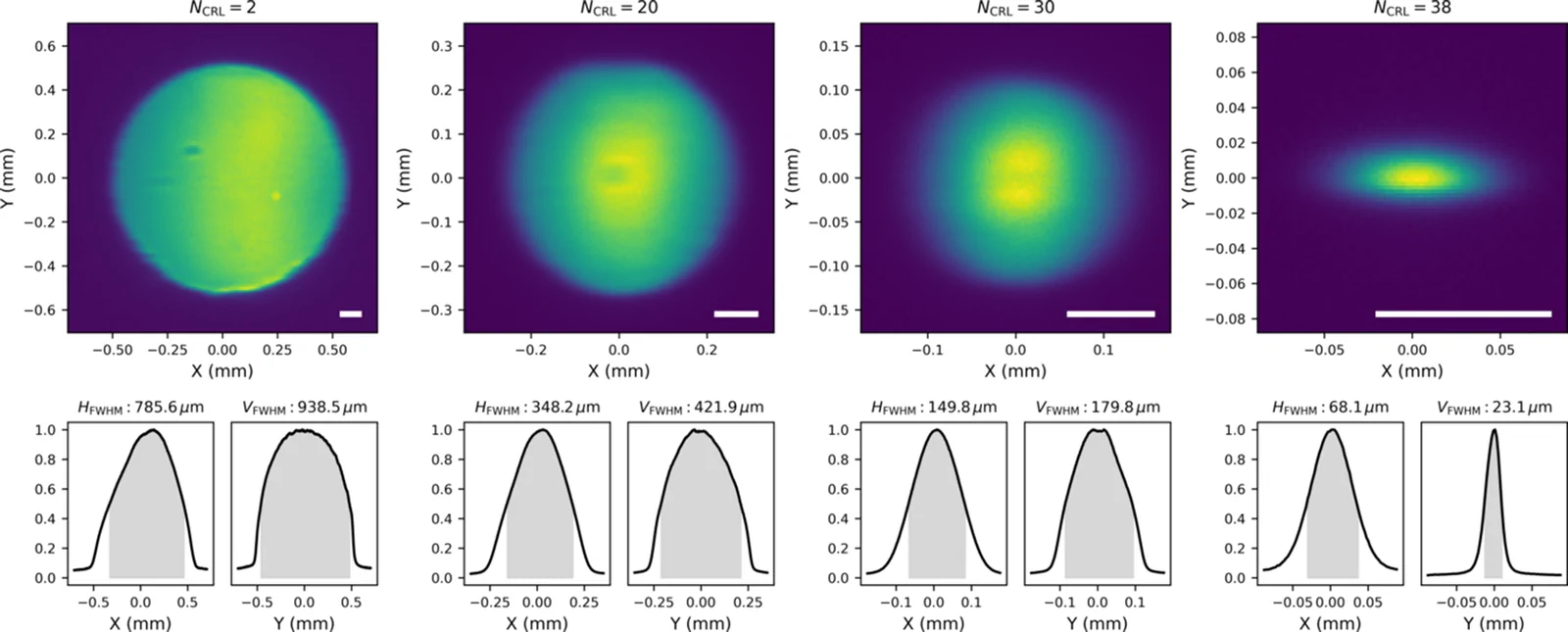
Examples of beam profiles with 2, 20, 30, and 38 CRLs inserted at 35.00 keV recorded with a beam viewer on the PXRD2D sample position (13.4 m from the CRLs). The top panels show the intensity distribution on a normalized linear color scale. The scale bars are 100 µm in all images. The images are magnified by a factor of two for every step from left to right (increasing number of CRL). The beam profiles below show the normalized intensity profiles either horizontally or vertically over the whole image. The gray shaded area shows the FWHM.

Focusing using the CRLs is performed in seconds using control system functions that estimate the number of lenses needed for a specific beam size and photon energy. The beam size can be verified using either a beam viewer on (or near) the sample position, or a knife edge scan. Examples of beam profiles are shown in Fig. 5 ▸. Measured flux and focused beam sizes are listed in Table 3 ▸.

**Figure 6:**
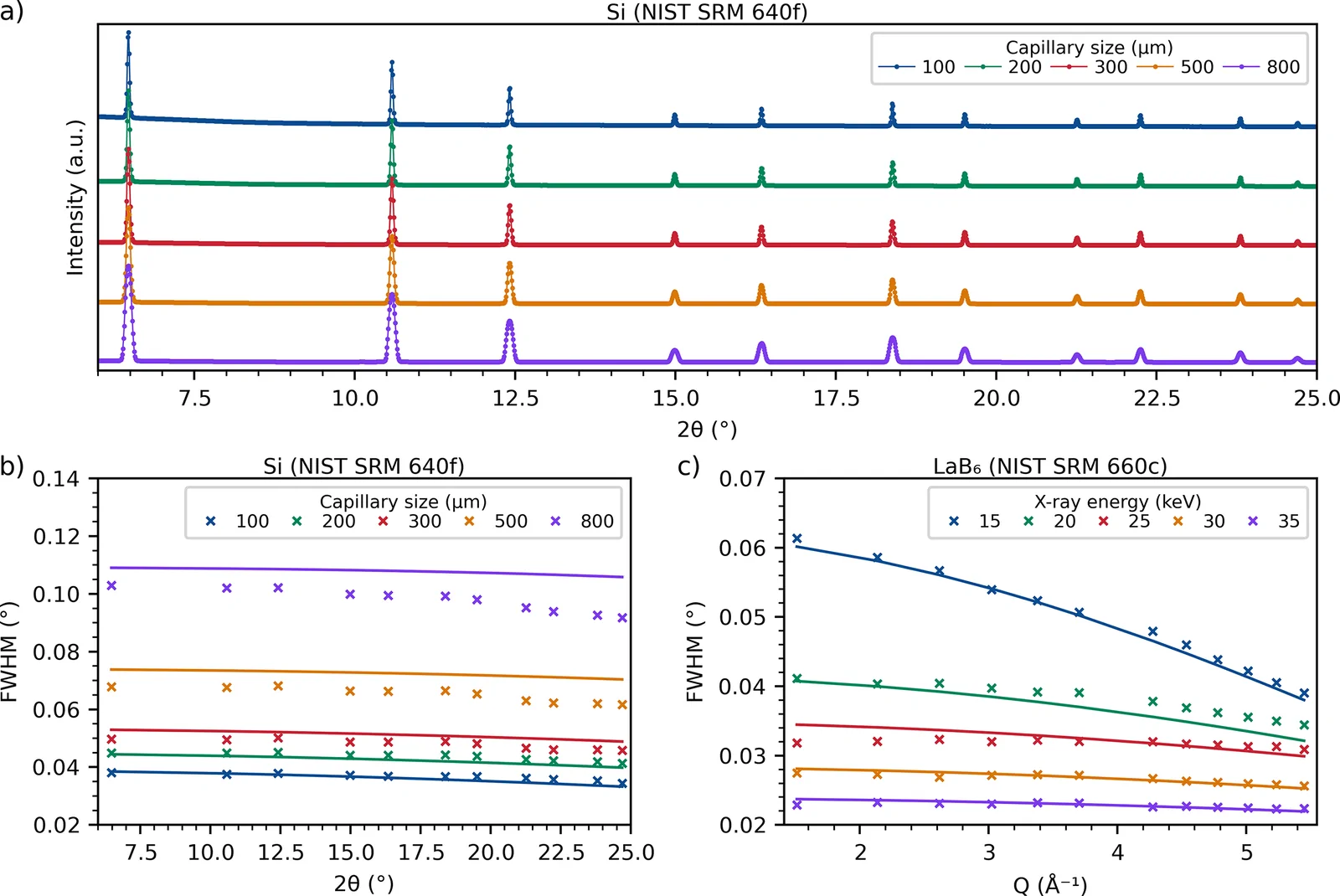
Comparison of diffraction patterns and fitted peak FWHM values obtained from diffraction powder standards at different conditions (Si NIST SRM 640f and LaB_6_ NIST SRM 660c). (*a*) Diffraction patterns of Si powder packed in capillaries with varying diameters; SDD: 350 mm; energy: 35.00 keV. (*b*) Single-peak fit FWHM values of Si diffraction patterns (symbols) and expected resolution (line); SDD: 350 mm; energy: 35.00 keV. (*c*) Single-peak fit FWHM values of LaB_6_ powders measured at varying distances and energies, keeping the *Q*_max_ constant (∼5.7 Å^−1^) and expected resolution (line); capillary size: 100 µm.

The instrument resolution of the powder diffractometer is best described using measurements of known standards with low to negligible sample broadening, here Si powder (SRM 640f, NIST, USA) and LaB_6_ (SRM 660c, NIST, USA). The resolution, expressed as the full width at half-maximum (FWHM) of individual peaks, depends on many parameters such as sample-to-detector-distance (SDD), pixel size, X-ray energy, energy resolution, beam divergence, and scattering volume (convolution of the beam size and sample size). The dominating factor is the beam footprint on the detector, which in turn depends primarily on the SDD and scattering volume. The effect of capillary size on the peak widths of Si diffraction peaks is shown in Fig. 6 ▸(*a*), where the broadening is readily visible as the capillary diameters increase from 100 µm up to 800 µm. This is further quantified in Fig. 6 ▸(*b*), which shows the peak FWHM values obtained from single-peak fitting. The expected instrumental resolution can be reasonably estimated with a modified implementation of the resolution function introduced by Chernyshov *et al.* (2021 ▸), shown as solid lines in Figs. 6 ▸(*b*) and 6(*c*). The resolution function can be estimated for different detectors, detector geometries, energies, and scattering volumes using *xrdPlanner* (Krause *et al.*, 2024 ▸).

Fig. 6 ▸(*c*) compares the fitted FWHM values for LaB_6_ peaks from diffraction patterns measured at varying energies and distances but with similar *Q*_max_ (∼5.7 Å^−1^). The difference in peak widths is immediately apparent, with the most instrumental broadening at low-energy/short-distance (15.00 keV/∼200 mm) and the least instrumental broadening at high-energy/long-distance (35.00 keV/∼570 mm), less than half of the peak widths of the low energy case. The instrumental broadening furthermore varies less as function of *Q* for the high-energy/long-distance case.

**Figure 7:**
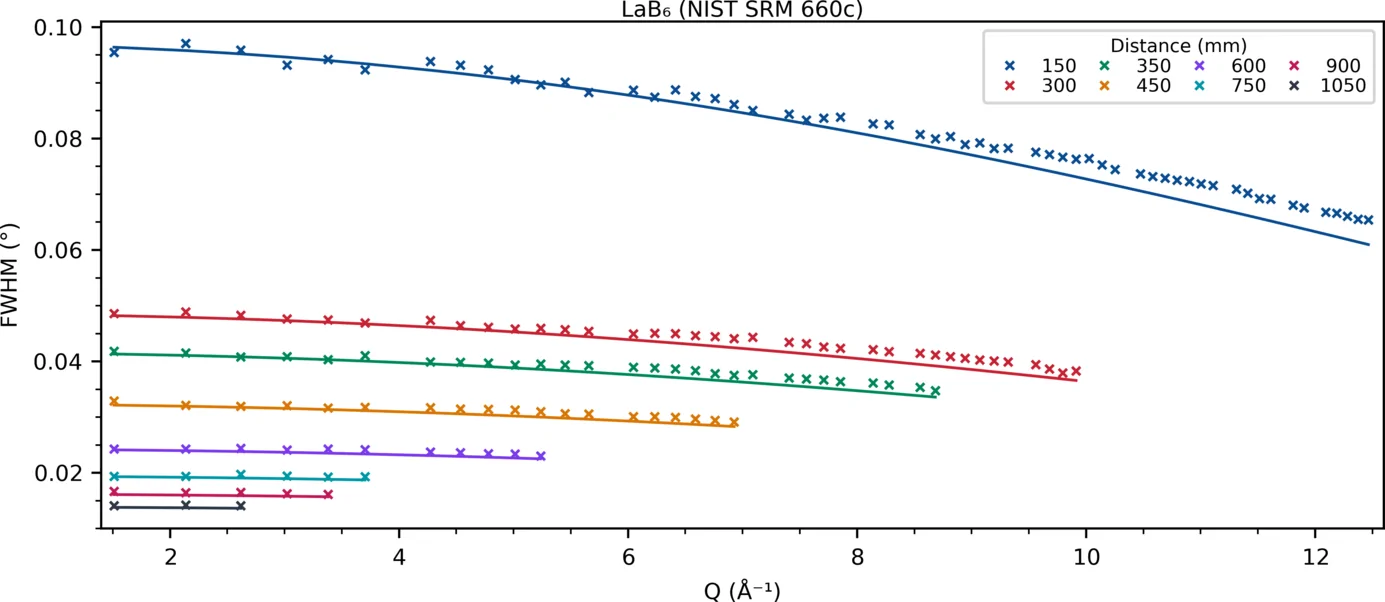
Single-peak fit FWHM values of LaB_6_ powder (symbols) and expected resolution (line) at varying distances. Capillary size: 100 µm; energy 35.00 keV.

In terms of instrumental resolution, the best results are achieved at high energy (35.00 keV) and higher SDD, combined with a sample as small as reasonably achievable. In practice, it is always a trade-off between several factors, such as resolution, count rate, sample (and sample environment) constraints, and *Q*-range, see Fig. 7 ▸. Furthermore, azimuthal range (ring coverage) might also factor into the considerations, *e.g.* in experiments involving crystallographic texture or strain where the Debye–Scherrer rings are no longer uniform. Such cases are hard to generalize, as they are often sample dependent.

## 6. Experimental highlights

Representative examples of recent results from DanMAX are given in this section.

### 6.1. 2D µPXRD and µXRF mapping

**Figure 8:**
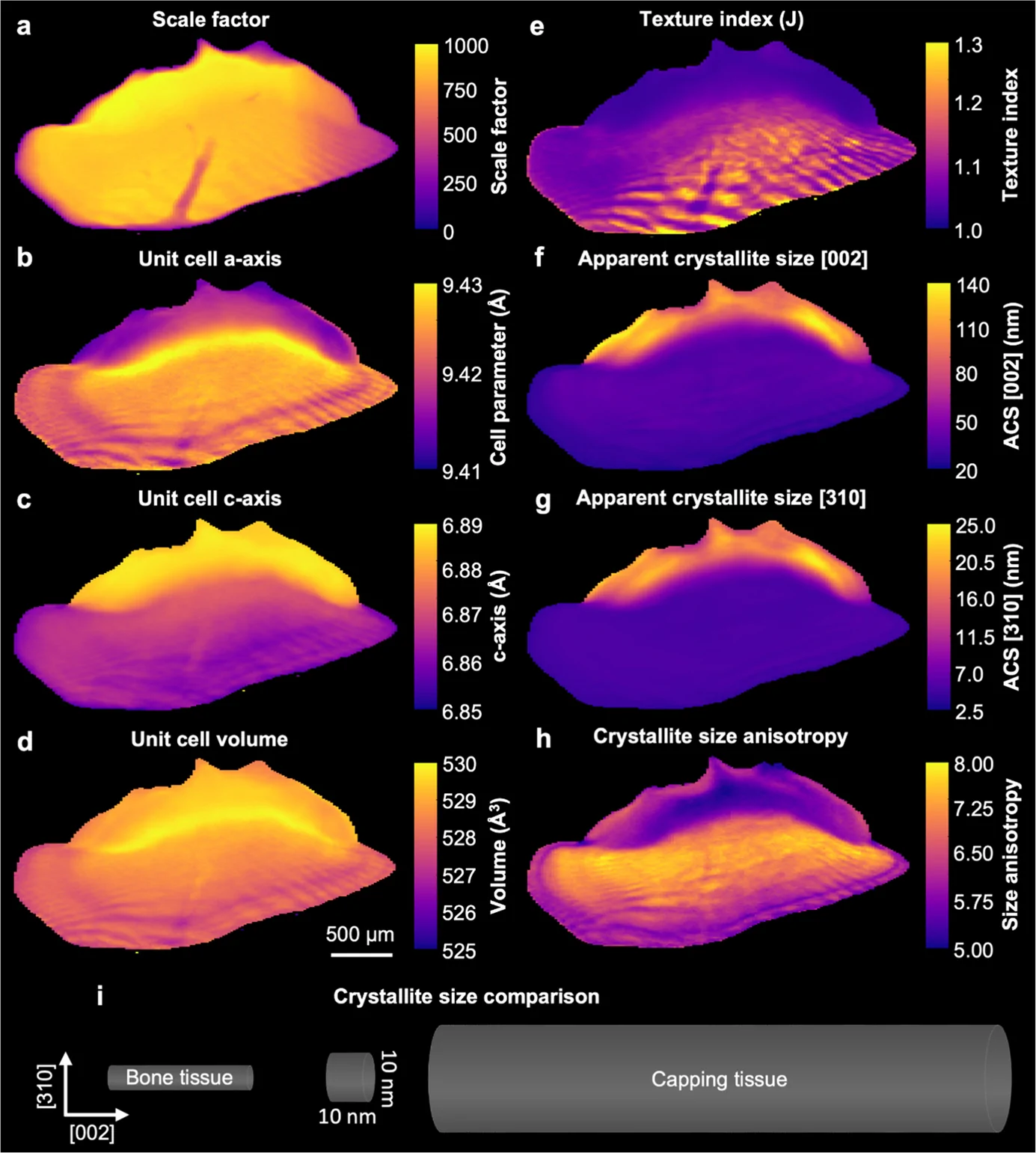
Scanning X-ray diffraction of an osteoderm. (*a*)–(*e*) Rietveld refined crystallographic parameters for a hy­droxy­apatite model: (*a*) scale factor, (*b*) unit cell *a*-axis, (*c*) unit cell *c*-axis, (*d*) unit cell volume, and (*e*) texture index. (*f*)–(*h*) Apparent crystallite dimensions calculated from the refined peak positions and FWHM. Reprinted from Rodriguez-Palomo *et al.* (2025 ▸) under a CC BY 4.0 License.

The combination of an intense, focused X-ray beam and high-speed continuous scanning at the beamline enables simultaneous mapping of crystallographic structure and elemental composition over large areas with micrometre-scale resolution. Using a beam focused to 43.9 µm × 15.0 µm (H × V, FWHM) and raster scanning in 20 µm × 10 µm steps at 80 Hz (corresponding to an X-ray exposure time of 11.5 ms per point), Rodriguez-Palomo *et al.* (2025 ▸, 2026 ▸) employed this approach to study mineralized osteoderms from the Mexican beaded lizard (*Heloderma horridum*), see Fig. 8 ▸, and other lizard species. The µPXRD provided spatially resolved diffraction patterns suitable for automated full Rietveld refinement (Frølich & Birkedal, 2015 ▸), allowing extraction of unit-cell parameters, crystallite size, and texture, while µXRF delivered quantitative maps of key elements such as Ca, Sr, and Zn.

These experiments revealed that the capping tissue, a mineralized superficial layer, differs fundamentally from underlying bone. In *H. horridum*, the capping tissue achieves enamel-like stiffness (elastic modulus up to ∼100 GPa) despite an almost isotropic arrangement of large hy­droxy­apatite crystals (lengths > 100 nm), a structural strategy distinct from the highly textured organization of the large hy­droxy­apatite crystals seen in enamel. Strontium enrichment and a graded transition zone between bone and capping tissue were also identified; features likely contributing to mechanical performance and stress dissipation (Rodriguez-Palomo *et al.*, 2025 ▸). Comparative studies across six species demonstrated striking diversity: while *H. horridum* and *Broadleysaurus major* share large, poorly oriented crystals and extreme stiffness, other species exhibit smaller, highly textured crystals and more moderate mechanical properties (Rodriguez-Palomo *et al.*, 2026 ▸). These insights were possible through the high spatial resolution and rapid acquisition offered by DanMAX, which enabled correlative mapping of structure, chemistry, and mechanics across entire osteoderms. We have since developed methods for scanning irregular areas to avoid scanning the area around non-rectangular samples like these (Gjørup *et al.*, 2026 ▸).

### 6.2. Batteries

Batteries are ubiquitous in our technology today and are poised to have an even larger role in the future as various applications transition from fossil fuels to electric energy. The mechanisms of (de)li­thia­tion in battery materials have been and remain an important field of study. Understanding the degradation mechanisms is essential to improving cell cycle life, and here it has been shown that spatial inhomogeneity strongly influences battery performance. Graae *et al.* (2023 ▸) demonstrated how fast scanning combined with *in operando* PXRD could be used to probe the Li inventory in prototype batteries with a silicon and graphite (Si–Gr) anode and an NMC811, low Co, cathode. Maps of 610 diffractograms, 10 rows of 61 points, were recorded every 12 min during charge and discharge, corresponding to 72 full 2D maps during a full charge/discharge cycle. From the PXRD data it was observed that the state of charge (SOC) inhomogeneity increased significantly with degradation in the Si–Gr cells, with up to 20% SOC difference across the graphite in the anode, and 5–6% SOC variation across the cathode, despite the cathode itself not being very degraded. The flexibility of the X-ray optics was utilized by Christensen *et al.* (2023 ▸) conducting a systematic study of the effect of the X-ray beam on the battery chemistry during *in operando* experiments. Half-cells were assembled in AMPIX cells (Borkiewicz *et al.*, 2012 ▸) and charged under various beam energies and exposures, emulating *in operando* experiments. Following this, the battery stack was removed from the cells, the beam energy changed, the beam was focused, and subsequently used to map out the spatially resolved diffraction signal. Here, it was revealed that the beam damage not only depends on both the X-ray energy and the amount of exposure but also depends strongly on the cell chemistry.

### 6.3. *In situ* PDF and PXRD during nanoparticle synthesis

TS experiments are normally conducted at high-energy beamlines to access high-momentum transfers necessary to avoid ripples in the reduced PDF originating from truncation errors. However, in *in situ* experiments, where the exposure time is on the order of seconds, truncation of the data is often necessary between 15 to 20 Å^−1^. The PDF can, despite the lower *Q*_max_, still yield important structural insight into the process. The PXRD2D instrument was constructed to collect scattering data up to ∼18 Å^−1^, *i.e.* comparable with the practically attainable resolution in many *in situ* experiments; however, using 35 keV X-rays. The scattering cross-section scales as the wavelength cubed and is thus much stronger at 35 keV compared with the higher energy beamlines. This, combined with the virtually noise-free hybrid pixel array detector, has been used to demonstrate the feasibility of usable PDF data acquisitions up to 250 Hz (Magnard *et al.*, 2023 ▸; Roelsgaard *et al.*, 2023 ▸).

**Figure 9:**
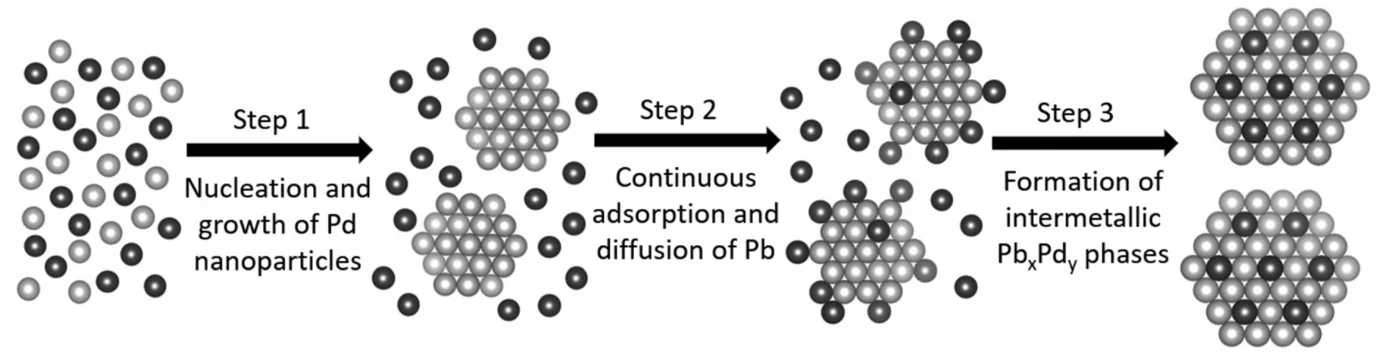
Illustration of the proposed mechanism for the formation of intermetallic Pb*_x_*Pd*_y_* nanoparticles. The light gray atoms are Pd, while the dark gray atoms are Pb. For simplicity, ions and atoms are shown by the same symbols, and counterions are omitted. Reproduced from Borup *et al.* (2023 ▸) with permission from the Royal Society of Chemistry.

To obtain the required scattering angles, it is necessary to place the detector very close to the sample, which causes broadening of the diffraction peaks due to the finite pixel size of the detector (see above), which in turn dampens the PDF (Chernyshov *et al.*, 2024 ▸). This means that this instrument is not well suited for studying highly crystalline samples using PDF; it is, however, quite capable for nano-crystalline and amorphous samples, as demonstrated for example, by systematic development of multi-metal nanoparticle electrocatalysts (Broge *et al.*, 2023 ▸; Borup *et al.*, 2023 ▸; Bertelsen *et al.*, 2024 ▸; Bertelsen *et al.*, 2025 ▸). In these studies, *in situ* PXRD and PDF are used to identify specific conditions for the solvothermal synthesis of advanced catalyst materials, such as Pt−Ir−Pd−Rh−Ru−Cu−Ni−Co high entropy alloys (Broge *et al.*, 2023 ▸), which are then reproduced at scale in the home laboratory. Importantly, the *in situ* studies not only accelerate materials discovery but also provide atomistic insight into the nanocrystal formation mechanism, as shown in the case of Pd_*x*_Pb_*y*_ intermetallic nanoparticles, where Pd nanocrystal seeds precipitate initially, followed by Pb diffusion to create the intermetallic crystal (Borup *et al.*, 2023 ▸), see Fig. 9 ▸. In the case of high-performance Ir_1–*x*_Ru_*x*_O_2_ nanoparticles for the oxygen evolution reaction, *in situ* PDF analysis revealed metal mixing already in the molecular precursor clusters as a prerequisite for the synthesis of the full composition range and thereby the ability to balance activity and stability effects in the catalyst (Bertelsen *et al.*, 2024 ▸).

### 6.4. Transformation-induced plasticity in steel

Understanding the mechanical behavior of metastable steels under dynamic loading is essential for applications requiring high strength and ductility. These alloys exhibit transformation-induced plasticity (TRIP), where the parent austenite phase transforms into martensite during deformation, enhancing strain hardening and energy absorption. However, this transformation is highly sensitive to strain rate, temperature, and stress state, making real-time characterization critical. DanMAX has enabled such investigations combining time-resolved synchronized X-ray diffraction, mechanical testing, optical, and infrared imaging.

**Figure 10:**
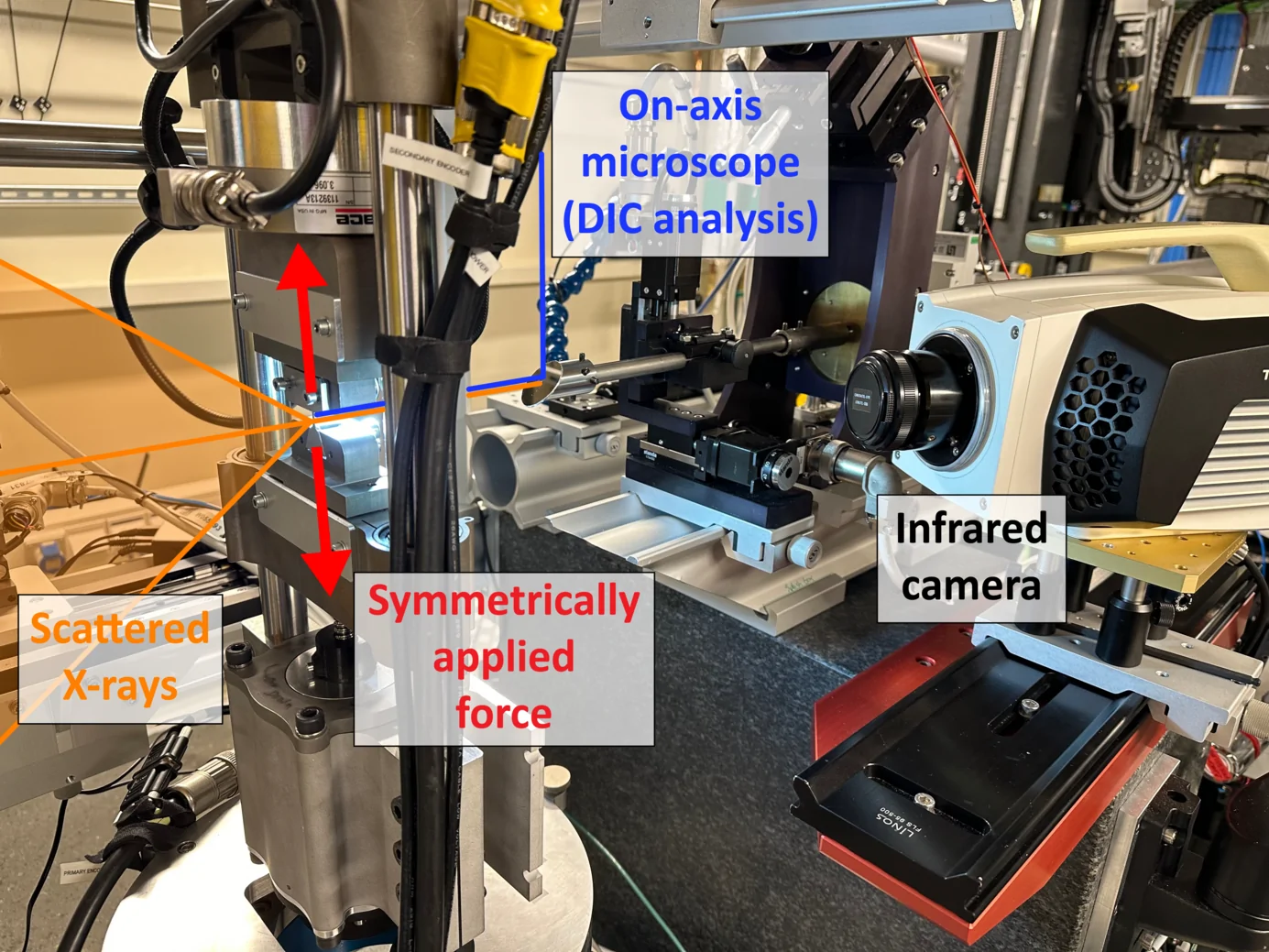
Photograph showing the experimental setup on the PXRD2D instrument. The tensile testing rig pulls the sample in the vertical direction with symmetric motion of the jaws (red arrows). The X-ray beam (orange line) propagates from right to left. Optical images are collected with an on-axis microscope (blue lines), and infrared images are recorded with an off-axis IR camera.

These studies have demonstrated that increasing strain rate from 10^−3^ s^−1^ to 1 s^−1^ significantly suppresses the γ→α′ martensitic transformation due to adiabatic heating and the associated rise in stacking fault energy. For metastable austenitic stainless steel EN 1.4318, the α′-martensite fraction decreased from approximately 0.86 at 10^−3^ s^−1^ to 0.26 at 1 s^−1^ at the onset of necking (Pun *et al.*, 2024 ▸; Isakov *et al.*, 2023 ▸). Similar trends were observed in TRIP-assisted multiphase steels (DH800 and TRIP700), where the transformation rate was highest at 10^−1^ s^−1^ and lowest at 1 s^−1^, with most martensite forming immediately after yielding (Langi *et al.*, 2025 ▸). Grain orientation also played a decisive role: 〈100〉γ fibers transformed preferentially at low strain rates, while 〈111〉γ fibers required higher stress and strain and were increasingly suppressed at elevated strain rates (Pun *et al.*, 2024 ▸). The integration of a custom mechanical rig, hardware-synchronized optical digital image correlation, infrared thermography, and diffraction demonstrates the beamline’s flexibility and experimental possibilities. The setup is shown in Fig. 10 ▸.

## 7. Conclusion and future development

DanMAX is a hard X-ray beamline for materials science that employs powder X-ray diffraction, total scattering, and full-field imaging. The source and optics have been designed to be easy to use and provide high flux in the energy range from 15 to 35 keV. By utilizing compound refractive lenses, it is fast and easy to focus the beam onto the sample.

The endstation instrumentation has been developed to allow a wide range of *in situ* and *in operando* experiments. The first of the three planned instruments, a powder diffractometer utilizing an area detector, has been described here. The imaging instrument has been installed and is now available in the user program. It will be described in detail in a future paper. A third instrument, a high resolution powder diffractometer, based on the MYTHENIII strip detector (Andrä *et al.*, 2025 ▸), has been procured and is being installed at the time of writing.

Significant effort has been invested in providing rapid and relevant feedback to the users/operators of the beamline. Diffraction images are shown live on the control computer, along with azimuthally integrated data. A large portfolio of scripts allows for online processing and visualization of the data during a beam time to feedback into the ongoing experiment. All processing is performed on MAX IV computers via a web interface, allowing team members off-site to participate in the data analysis.

MAX IV was the first operational fourth-generation synchrotron source and is currently planning an upgrade, MAX 4^U^, decreasing the emittance from the 328 pm rad today to below 75 pm rad. This will decrease the horizontal source size further, yielding a more symmetric focused beam with higher flux density. The higher flux density and smaller, more symmetric beam will significantly improve, for example, the 2D and 3D mapping techniques described above.

## Figures and Tables

**Table 1:** Specification of the DanMAX beamline

Photon source	
Electron beam size, σ (H × V)	53.9 µm × 6.4 µm (RMS)
Electron beam divergence, σ′ (H × V)	11.3 µrad × 9.8 µrad (RMS)
Length	3 m
Period	16 mm
Maximum deflection parameter (*K*)	1.66
Phase error	≤2.5°†
Total power (4 mm)	10.4 kW

High pass filter	Single crystal diamond, CVD, 1 mm
Distance from source	23.5 m

Double crystal monochromator (DCM)	
Distance from source	27.1 m
Lattice	Si(111)
Deflection	Horizontal (4 or 10 mm)
Band-pass (Δ*E*/*E*)	1.7 × 10^−4^ @ 15 keV to 3.2 × 10^−4^ @ 35 keV‡
Cooling	Liquid N_2_
Energy range	12–35 keV

Double multilayer monochromator (MLM)	
Distance from source	28.3 m
Materials	[W/B_4_C]_200_ @ Si(100) / [Ni_93_V_7_/B_4_C]_400_ @ Si(100)
*d*-spacing and thickness ratio, Γ	25 Å, 0.8 / 20 Å, 0.65
Deflection	Horizontal (6 or 10 mm)
Band-pass (Δ*E*/*E*)	0.8 × 10^−2^ @ 35 keV / 0.4 × 10^−2^ @ 35 keV†
Cooling	Water
Energy range	12–35 keV

Transfocator	
Distance from source	30.9 m
Distance to imaging sample position	9.1 m
Distance to PXRD2D sample position	13.5 m
Lens material	Be (O30H)
Lens radius and geometrical aperture	200 µm, 880 µm
Number of lenses	50

† Measured.

‡ Simulated.

**Table 2:** List of available stages and sample environments Some sample environments are compatible with more of the endstations at DanMAX; these are listed in the right-hand column. µCT is the full-field imaging instrument. HERDi is a high resolution PXRD instrument currently under construction.

Type	Description	Endstation
Capillary spinner	Horizontal or vertical spinning axis. Manual goniometer head	PXRD2D
µXRD + XRF mapping	*XY* linear stages	PXRD2D
XRD-CT	Rotary stage on linear stage	PXRD2D, µCT
Diamond anvil cell	XY stage on rotary stage for DAC alignment	PXRD2D
Cryostream: 80 K to 500 K	Oxford Cryosystems – Cryostream 800 Plus	PXRD2D, µCT, HERDi
Hot air blower: RT to 1000 K	HyBec Heater	PXRD2D, µCT, HERDi
Very fast heating rates	ARΩS furnace	PXRD2D
Potentiostat	Biologic VMP3 – 14 channels	PXRD2D, µCT
Solvothermal reactor	Capillary reactor	PXRD2D
Gas regulator: 0 to 200 bar	Druck Pace 5000; inert gases	PXRD2D, µCT
Gas mixing panel: 0 to 200 bar	Mixing of inert gases and CO_2_	PXRD2D, µCT
Humidifier	Cellkraft, P-2	PXRD2D, µCT

**Table 3:** Measured flux and beam size (at the PXRD2D instrument) as a function of photon energy in keV and monochromator type Beam size is given as FWHM in µm. All flux values are in 10^12^ photons s^−1^.

	Full beam, ∼1000 µm × 1000 µm			Slitted beam, 500 µm × 500 µm			Focused beam					
	DCM	MLM		DCM	MLM		DCM		MLM			
	Si(111)	W	Ni	Si(111)	W	Ni	Si(111)		W		Ni	
Energy	Flux	Flux	Flux	Flux	Flux	Flux	Size	Flux	Size	Flux	Size	Flux
15	18	190	110	2.5	16	9.5	60 × 17	7.1	67 × 22	65	134 × 22	34
25	5.1	150	81	1.0	13	9.4	82 × 22	2.0	70 × 24	41	132 × 24	27
35	1.3	52	27	0.2	5.6	2.9	64 × 19	0.4	77 × 26	11	108 × 19	6.8
